# Association Between Neonatal and Maternal Vitamin D Levels at Birth

**DOI:** 10.7759/cureus.72261

**Published:** 2024-10-24

**Authors:** Satvik Jaiswal, Utkarsh Bansal, Ekansh Rathoria, Richa Rathoria, Ravindra Ahuja, Anjana Agarwal

**Affiliations:** 1 Pediatrics, Hind Institute of Medical Sciences, Barabanki, IND; 2 Pediatrics, Hind Institute of Medical Sciences, Sitapur, IND; 3 Obstetrics and Gynecology, Hind Institute of Medical Sciences, Sitapur, IND; 4 Obstetrics and Gynecology, Hind Institute of Medical Sciences, Barabanki, IND

**Keywords:** cord blood vitamin d levels, hypovitaminosis d, infant’s nutrition, mother-newborn dyads, neonatal vitamin d deficiency, newborn infant, serum 25(oh)d concentration, vitamin d deficiency, vitamin d insufficiency, vitamin d supplementation

## Abstract

Background

Vitamin D is an important nutrient for skeletal and extra-skeletal health. Mothers and their neonates are frequently vitamin D deficient. This study aimed to find the association of neonatal vitamin D levels with maternal vitamin D levels at birth.

Materials and methods

This descriptive-observational study was done on mother-baby dyads at a tertiary center, which included mothers delivering a healthy baby at term gestation. In this study, we reviewed 102 mother-baby dyads. The maternal venous blood and cord blood samples were collected after delivery to determine vitamin D levels. Data collected were maternal socio-demographic variables, weight, height, and neonatal anthropometric variables. The descriptive statistics, chi-square test, and Pearson’s R were used for analysis. The significant p-value was <0.05.

Results

The mean (SD) age and body mass index (BMI) of the mothers were 26.50 (4.04) years and 24.27 (4.06) kg/m^2^, respectively. Vitamin D insufficient levels were reported in 96 (92.2%) mothers and 100 (98%) neonates. The mean (SD) vitamin D levels of the mother and neonate were 16.20 (8.29) and 15.23 (7.06) ng/mL, respectively, and were positively associated (chi-square value 3584.16; Pearson’s R-value 0.676; p<0.0001). A significant association was found between maternal age, maternal dressing type, BMI, and neonatal anthropometric variables with both maternal and neonatal vitamin D levels.

Conclusion

A high prevalence of vitamin D deficiency in both mothers and their neonates was found along with a positive correlation between their vitamin D levels. Diagnostic screenings for vitamin D levels and supplementation during pregnancy should be considered to prevent deficiency in the mother-baby dyad.

## Introduction

Bone mineral metabolism is dependent on vitamin D, which stimulates intestinal absorption of calcium (Ca) and phosphorus (P). The calcium-phosphorus product determines the mineralization of the collagen matrix, and its insufficiency can lead to osteomalacia in adults and rickets in children [1]. The non-classical actions of vitamin D, including the functioning of the immune, respiratory, and endocrine systems, have generated exponential interest in the scientific world, particularly in the role of vitamin D in the regulation of immunity [2]. Vitamin D receptor is expressed by cells of both the innate and adaptive immune systems [3]. Despite being a vital nutrient for human health, vitamin D deficiency is widespread both in children and adults. Globally, chronic vitamin D deficiency is an important childhood problem leading to rickets. Nutritional rickets is commonly seen in countries of Asia and the Middle East due to vitamin D deficiency, while in many countries of Africa, it is due to calcium deficiency. Though rickets is less common in developed countries due to regular infant supplementation, it is still prevalent in immigrants, refugees, and populations with darker skin [4]. This is especially true for nursing infants who are neither exposed adequately to sunlight nor given vitamin D supplements [5].

Vitamin D occurs naturally as cholecalciferol (vitamin D3) and ergocalciferol (vitamin D2) derived from animal and plant sources, respectively, which can be obtained from dietary sources, although only a few foods contain significant quantities [6]. Ultraviolet B irradiation (at wavelengths between 290 and 315 nm) in sunlight converts 7-dehydrocholesterol present in human skin layers, mainly the epidermis, to pre-vitamin D3. The unstable pre-vitamin D3 is eventually thermo-converted into vitamin D3 and on prolonged sun exposure into lumisterol and tachysterol, which are inactive solar photoproducts, which is the natural way to prevent solar vitamin D intoxication [6]. Vitamin D3 is transported to the liver and hydroxylated to produce 25-hydroxyvitamin D (25(OH)D), which is further hydroxylated into 1,25-dihydroxyvitamin D (1,25(OH)2D) or calcitriol, the bioactive form, in the kidney [6].

The prevalence and magnitude of vitamin D deficiency depend on the definition used in the reported studies. The Endocrine Society categorizes <20 ng/mL of serum 25(OH)D as vitamin D deficiency, which is accompanied by a persistent increase of parathyroid hormone (PTH) and a reduction in intestinal calcium absorption [7]. The ideal protective range for blood 25(OH)D levels is between 30 and 100 ng/mL because at levels of 30 ng/mL or above, intestinal calcium absorption peaks, and PTH levels continue to decline [4]. The Institute of Medicine (IOM) categorizes 25(OH)D concentrations <12 ng/mL while the Endocrine Society (ES) categorizes 25(OH)D concentrations <20 ng/mL as vitamin D deficiency [7,8].

The tropical Indian subcontinent has a high prevalence of vitamin D insufficiency, which varies from 50% to 94% in various studies [9]. Vitamin D deficiency was reported in 44.3-66.7% of infants, 84.9-100% of school-age children, 42-74% of pregnant females, 70-81.1% of breastfeeding mothers, and 30-91.2% of adults [10]. A recent study from Rishikesh by Chacham S et al. (2020) reported hypovitaminosis D in 74% of the study infants with major determinants of deficiency as maternal vitamin D deficiency, neonatal age group, and lower socioeconomic status [11]. Another study from Nepal by Shrestha D et al. (2019) reported vitamin D deficiency in 81% of mothers and 35.8% of their babies [12]. Vitamin D deficiency in unsupplemented infants was reported to be 24% in Argentina, 40% in Australia, 46% in America, 69% in Germany, and 99% in India [13]. Serum 25(OH)D levels below 12 ng/mL were linked to a higher risk of developing nutritional rickets [5].

The benefits of breastfeeding are unquestionable because breast milk contains almost all the necessary nutrients for the proper growth of an infant. The infant's daily requirement of 400IU vitamin D is not met by breast milk (20-70 IU/L) alone, especially if the mother is vitamin D deficient and exposure to sunlight is restricted [5]. The Indian Academy of Pediatrics recommends 400 IU/day of vitamin D supplementation during infancy [14]. A recent study by Rabbani S et al. (2021) reported a significant vitamin D deficiency in pregnant women and their babies, as well as a positive association between maternal and neonatal 25(OH)D levels [15]. In this context, the present study was conducted to estimate the association between neonatal and maternal vitamin D levels at birth.

## Materials and methods

Study settings

This descriptive observational study was on all mother-baby dyads delivered at a tertiary care center over 15 months, from September 2021 to November 2022, to estimate the association between neonatal and maternal vitamin D levels at birth after obtaining informed consent from the parents. The study was approved by our institution’s ethics committee.

We included mothers delivering a term (37 to 41(+6) weeks) singleton newborn. We excluded mothers a) with underlying diseases of the liver, kidney, or heart; b) with chronic illnesses like tuberculosis, diabetes mellitus, severe anemia, HIV, hepatitis B, gestational diabetes mellitus, pregnancy-induced hypertension, photosensitivity, and hypothyroidism; c) taking drugs influencing vitamin D metabolism (antiepileptic drugs, corticosteroids, statins, antitubercular drugs, antiretroviral drugs, histamine H2-receptor antagonists, thiazides, and chemotherapeutic drugs [16]); d) whose neonates were born with low birth weight (<2500 g), had moderate to severe perinatal asphyxia (first minute Apgar score of <7), required immediate neonatal intensive care unit admission, and were born with known chromosomal abnormalities, or congenital malformations.

Sampling

Using OpenEpi online software (www.OpenEpi.com), the sample size was calculated to be 98 mother-newborn dyads by considering 54.5% of the mothers had severe vitamin D deficiency from a previous study, the power of the study as 80%, and an allowable error of 5% [11]. The final sample recruited was 102 mother-newborn dyads. The convenience sampling method was used.

Measurements

At the time of recruitment, the maternal venous blood and cord blood samples were collected after delivery; serum was separated and stored at -20°C for an estimation of 25(OH)D concentration. The 25(OH)D concentration was determined by the chemiluminescence immunoassay (CLIA) method (Access2 kits of Beckman Coulter, Brea, California, US). In this study, we employed the International Endocrine Society guidelines to categorize serum 25(OH)D levels as vitamin D deficiency at level <20 ng/mL, insufficiency at 21-29 ng/mL, and sufficiency at ≥30 ng/mL [7]. Also, the serum levels of calcium and phosphorous were investigated.

Data collection and analysis

Data were collected on a predesigned proforma, which included socio-demographic variables. The body mass index (BMI) of the mother was calculated by weight in kilograms (kg) divided by her height in meters (m) squared. The gestation age was measured in weeks, from the first day of the last menstrual period to birth. Neonates were thoroughly examined after birth and gestation age was reassessed by Modified Ballard’s scoring. The weight, length, head circumference, chest circumference, and area of the anterior fontanelle of neonates were measured at birth as per standard methods and noted.

The descriptive statistics, chi-square test, and Pearson’s R were used to find any association or correlation between 25(OH)D concentrations of mothers and neonates using SPSS software version 26 (IBM Corp. Armonk, NY, US). A p-value of <0.05 was considered significant.

## Results

In this study, the majority of the mothers (89; 87.3%) were <30 years of age, 73 (71.6%) were Hindu by religion, 76 (74.5%) were vegetarian, 81 (79.4%) wore partially covered dressing, and 65 (63.7%) were primigravida (Table 1). The mean (SD) age of the mothers was 26.50 (4.04) years while the mean (SD) BMI of mothers was 24.27 (4.06) kg/m^2^.

**Table 1:** Socio-demographic variables of mothers (n=102) BMI= Body Mass Index

Variable		Number (n)	Percentage (%)
Age (years)	<25	53	52
	26-30	36	35.3
	31-35	8	7.8
	>35	5	4.9
Religion	Hindu	73	71.6
	Muslim	29	28.4
Diet	Vegetarian	76	74.5
	Non-vegetarian	26	25.5
Dressing	Partially covered	81	79.4
	Fully covered	21	20.6
Gravida	Primigravida	65	63.7
	Multigravida	37	36.3
BMI (kg/m^2^)	<25	64	62.7
	>25	38	37.3

Table 2 represents the mean and standard deviation of the anthropometric variables of neonates.

**Table 2:** Mean and standard deviation of anthropometric variables of neonates (n=102)

Variables	Mean	Standard Deviation
Weight (g)	2805	260
Length (cm)	48.83	2.44
Head Circumference (cm)	33.99	1.99
Chest Circumference (cm)	31.93	2.49
Anterior Fontanelle (cm^2^)	4.18	1.91

Vitamin D deficiency was observed in 67 (65.7%) mothers and 80 (78.4%) neonates, respectively, and insufficiency in 27 (26.5%) mothers and 20 (19.6%) neonates, respectively (Table 3).

**Table 3:** Categorization based on vitamin D levels in mothers and neonates (n=102)* *Number in parenthesis indicates percentages

	Vitamin D levels		
	Deficiency (<20 ng/mL)	Insufficiency (21-29 ng/mL)	Sufficiency (≥30 ng/mL)
Mothers	67 (65.7)	27 (26.5)	8 (7.8)
Neonates	80 (78.4)	20 (19.6)	2 (2)

The mean (SD) vitamin D levels of the mother and neonate were 16.20 (8.29) and 15.23 (7.06) ng/mL, respectively. The maternal and neonatal vitamin D levels were found to be significantly associated (p<0.05) (Table 4). The maternal and neonatal serum calcium and phosphorous levels were also found to be significantly associated (p<0.05).

**Table 4:** Association between maternal and neonatal vitamin D levels, serum calcium levels, and serum phosphorous levels (n=102) *SD= Standard Deviation, significant p-value <0.05

Variables	Mother	Neonate	Chi-square test value	p-value	Pearson’s R	p-value
	Mean (SD)	Mean (SD)				
Vitamin D Levels (ng/mL)	16.20 (8.29)	15.23 (7.06)	3584.16	<0.0001	0.676	<0.0001
Serum Calcium (mg/dL)	8.58 (0.87)	9.18 (0.74)	1329.75	<0.0001	0.245	0.013
Serum Phosphorus (mg/dL)	4.26 (0.88)	6.12 (1.07)	1537.86	<0.0001	0.216	0.029

**Figure 1:**
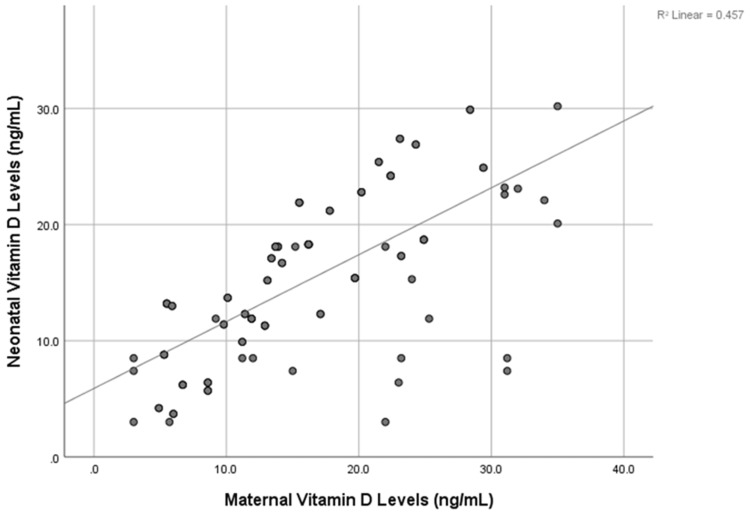
Correlation between vitamin D levels of mother and neonate (n=102)

A significant correlation was seen between maternal and neonatal vitamin D levels (Pearson R-value 0.676; p<0.0001) (Table 4, Figure 1).

Among the sociodemographic variables of the mother, a significant association was found between maternal age, type of dressing, and BMI with both maternal and neonatal vitamin D levels, while religion was significantly associated with only maternal vitamin D levels (p<0.05) (Table 5). Both maternal and neonatal vitamin D levels were significantly associated with neonatal birth weight, length, head circumference, chest circumference, and anterior fontanelle area (p<0.05) (Table 5).

**Table 5:** Association between maternal sociodemographic variables and neonatal anthropometric variables with maternal and neonatal vitamin D levels (n=102) *Significant p-value <0.05 BMI= Body Mass Index, HC= Head Circumference, CC= Chest Circumference, AF= Anterior Fontanelle

Variables		Maternal vitamin D levels		Neonatal vitamin D levels	
		Chi-square test value	p-value	Chi-square test value	p-value
Maternal Variables	Age (years)	978.91	<0.0001	1012.83	<0.0001
	Religion	63.75	0.042	53.84	0.071
	Diet	57.51	0.119	53.82	0.071
	Dressing	68.67	0.017	55.92	0.048
	Gravida	21.1	0.99	10.72	1.0
	BMI (kg/m^2^)	2318.12	<0.0001	2370.48	<0.0001
Neonatal Variables	Weight (kg)	1364.95	<0.0001	1409.23	<0.0001
	Length (cm)	1187.13	<0.0001	1176.1	<0.0001
	HC (cm)	995.5	<0.0001	995.6	<0.0001
	CC (cm)	802.41	<0.0001	797.59	<0.0001
	AF (cm^2^)	875.33	<0.0001	898.26	<0.0001

## Discussion

The fetus is entirely dependent on its mother for its vitamin D supply, and maternal deficiency can lead to deficiency in the newborn. Clinical studies by Amrein K et al. (2020) and Arora S et al. (2018) on pregnancy outcomes in vitamin D-deficient mothers have reported an increase in preeclampsia, cesarean section, gestational diabetes, preterm delivery, and low birth weight neonates [17,18].

This study reported vitamin D deficiency in 67 (65.7%) mothers and 80 (78.4%) neonates and a significant correlation between maternal and neonatal vitamin D levels (Pearson R-value 0.676; p<0.0001). Similar to our study findings, the Rabbani S et al. (2021) study reported vitamin D deficiency in 61.5% (131) of the mothers and 99.5% (212) of their newborns, respectively, and a positive association between their 25(OH)D levels (r=0.66; p<0.001) [15]. Similarly, Arora S et al. (2018) reported hypovitaminosis D in 86% (172) of maternal and 85% (170) of cord blood samples, with a high correlation between the vitamin D levels of the two (p=0.001, correlation coefficient r=0.84) [18]. A systematic review and meta-analysis revealed in 2022 that pregnant South Asian women have a very high prevalence of vitamin D deficiency, with the greatest rate of inadequacy reported from Pakistan (76%), followed by India (67%), Bangladesh (64%), and Nepal (14%). The vitamin D levels of mothers varied from 9 ng/mL to 24.86 ng/mL, with a mean (SD) of 16.37 (7.13) ng/mL [19].

In the present study, the mean (SD) serum 25(OH)D levels of the mothers and neonates were 16.20 (8.29) and 15.23 (7.06) ng/mL, respectively, which was comparable to the Arora S et al. study reporting maternal and cord levels as 12.5 (6.8) ng/mL and 12.3 (7.1) ng/ml, respectively [18]. A study done in Thailand by Ariyawatkul K et al. (2018) reported higher mean (SD) serum 25OHD levels of mothers and cord blood at 25.42 (8.07) and 14.85 (5.13) ng/mL, respectively [20].

In the present study, the mean (SD) age of the mothers was 26.50 (4.04) years and the majority of the mothers (89; 87.3%) were <30 years of age, which was aligned with a study on the prevalence of vitamin D deficiency in South Indian women by Ravinder SS et al. (2022), with a mean (SD) age of 25.69 (3.56) years and most (92; 92%) below 30 years of age [21].

Our study found a significant association between maternal age and vitamin D levels of mothers and infants. A similar association was observed by Yu CK et al. (2011) and Miller KM et al. (2021) [22,23]. This may be because women becoming pregnant at a younger age are usually nutritionally deficient and uptake of supplements is higher in older pregnant women [24]. In contrast, a Slovenian study by Treiber M et al. (2020) reported no significant association between maternal age and vitamin D levels in mothers and neonates [25].

The present study found a significant association between religion and maternal vitamin D levels. Also, maternal and neonatal vitamin D levels were significantly associated with maternal dressing. Studies by Owie E et al. (2018), Ates S et al. (2016), and van der Pligt P et al. (2018) have observed that women who wear a specific form of clothing that fully covers their body, due to cultural or religious purposes, substantially restrict sunshine exposure and have a significant degree of vitamin D insufficiency [26-28].

There was no significant association between vitamin D levels and maternal dietary type in our study. Likewise, both vegetarians and non-vegetarians equally suffered from vitamin D insufficiency as seen in the Redecillas-Ferreiro S et al. (2021) study in Spain [29]. There was no association of maternal and neonatal vitamin D levels observed with the gravida status of the mother, similar to a previous study by Sonowal R et al. (2021) [30].

In the present study, maternal and neonatal vitamin D levels were significantly associated with maternal BMI. This was in accordance with a previous study by Ravinder SS et al. that found that high maternal BMI at delivery was significantly associated with vitamin D deficiency [21].

In the present study, maternal and neonatal vitamin D levels were significantly associated with neonatal birth weight, length, head circumference, chest circumference, and anterior fontanelle area. Akin to our study, a study by Sonowal R et al. from Assam documented that vitamin D levels of mothers were significantly associated with neonatal birth weight (p<0.001), length (p=0.0001), head circumference (p=0.0003), and chest circumference (p=0.001) [30], in contrast, Rabbani S et al. found no such association [15].

Limitations

Due to the expensive testing needed, the study was constrained by the small sample size. The study did not evaluate factors like pre-pregnancy vitamin D levels, ethnicity, occupation, the color of the skin, socioeconomic status, and environment, which may contribute to vitamin D status. The findings have to be confirmed in larger, multi-centric studies that consider the confounding factors. Longitudinal studies during the period of gestation, evaluating maternal vitamin D levels at multiple times may provide a comprehensive picture and determine the optimal maternal requirement of vitamin D during pregnancy.

## Conclusions

Our study showed that despite living in a region with abundant sunshine, pregnant women and their neonates had inadequate serum 25(OH)D concentrations. Maternal age, dressing style (sunlight exposure), and BMI have significant influences on their own as well as their neonates' vitamin D levels. It may be inferred that vitamin D deficiency during pregnancy is quite prevalent in this region of India and has a significant association with neonatal hypovitaminosis D. Vitamin D supplementation may be recommended to pregnant women with vitamin D deficiency, and further research is advocated to determine the optimum requirements of vitamin D in pregnancy. Studies on the impact of hypovitaminosis D on neonatal health outcomes are recommended.
